# Deep learning classification of urinary sediment crystals with optimal parameter tuning

**DOI:** 10.1038/s41598-022-25385-x

**Published:** 2022-12-07

**Authors:** Takahiro Nagai, Osamu Onodera, Shujiro Okuda

**Affiliations:** 1 Department of Neurology, Brain Research Institute, Niigata University, 1-757 Asahimachi-dori, Chuo-ku, Niigata, 951-8585 Japan; 2 Center for Genomic Data Management, Niigata University Medical and Dental Hospital, 1-754 Asahimachi-dori, Chuo-ku, Niigata, 951-8520 Japan; 3 Medical AI Center, Niigata University School of Medicine, 2-5274 Gakkocho-dori, Chuo-ku, Niigata, 951-8514 Japan; 4 Division of Bioinformatics, Niigata University Graduate School of Medical and Dental Sciences, 2-5274 Gakkocho-dori, Chuo-ku, Niigata, 951-8514 Japan

**Keywords:** Bioinformatics, Urology, Machine learning

## Abstract

The examination of urinary sediment crystals, the sedimentary components of urine, is useful in screening tests, and is always performed in medical examinations. The examination of urinary sediment crystals is typically done by classifying them under a microscope. Although automated analyzers are commercially available, manual classification is required, which is time-consuming and varies depending on the technologist performing the test and the laboratory. A set of test images was created, consisting of training, validation, and test images. The training images were transformed and augmented using various methods. The test images were classified to determine the patterns that could be correctly classified. Convolutional neural networks were used for training. Furthermore, we also considered the case where the crystal subcategories were not treated as separate. Learning with all parameters except the random cropping parameter showed the highest accuracy value. Treating the subcategories together or separately did not seem to affect the accuracy value. The accuracy of the best pattern was 0.918. When matched to a real-world case, the percentage of correct answers was 88%. Although the number of images was limited, good results were obtained in the classification of crystal images with optimal parameter tuning. The parameter optimization performed in this study can be used as a reference for future studies, with the goal of image classification by deep learning in clinical practice.

## Introduction

Urine sediment examination is the microscopic examination of the precipitated components obtained by centrifuging urine. It is a useful test for the diagnosis of various systemic diseases and is also used as a screening test^1^. If abnormal components are detected, further detailed examinations, such as blood tests, radiographs of the urinary tract system, and ultrasound examinations are performed. Urine sediment examination is used in routine clinical examinations and is also used in developed countries in the U.S. and Europe. Patients can collect their own urine, which is much less invasive than blood testing, making it one of the least invasive tests available.

Accurate classification of urinary sediment crystals requires visual inspection under a microscope by a clinical laboratory technician. Methods have already been developed to analyze the active constituents in urine by cytometry using autoanalyzers^2^ or to image the tangible substances in urine and classify them using an analytical system^3^. However, the price of these devices is extremely high, and the equipment requires periodic calibration and the cost of purchasing test reagents is high. Currently, the conventional speculum method is considered essential. Despite the simplicity of the urine specimen preparation process, the process of distinguishing the components of the urine sediment is time-consuming, and the accuracy depends on the skill of the technician and the facility environment of the laboratory, and is subject to variation^4^.

Many components can be observed in urine sediment, which can be broadly divided into cellular and crystalline components. Bacteria can be observed under a microscope. It has been reported that classification of only three components (cells, bacteria, and calcium oxalate crystal) was successful, with 97% accuracy^5^. Studies have also compared various network models for the classification of cells, bacteria, and calcium oxalate^6^. Among the cellular components of the urine sediment, eight types of cellular components (erythrocytes, leukocytes, epithelial cells, crystals, urinary columns, mycelia, and epithelial nuclei) can be detected and classified in less than 100 ms, with an accuracy of over 80%^7^. Among the cellular components, erythrocytes and leukocytes are of great diagnostic significance, but the morphology of erythrocytes varies depending on the site of leakage from blood vessels in the kidney, and the shape of leukocytes is not consistent, making classification a problem^8^. A study that attempted to discriminate 10 components (bacteria, yeast, calcium oxalate, hyaline columns, mucus filaments, sperm, squamous cells, erythrocytes, leukocytes, and leukocyte clusters) showed 97% classification accuracy^9^.

However, for crystal components, even if the crystal names are the same, the apparent shapes are different^10^, requiring expert classification techniques and knowledge, and, to the best of our knowledge, there have not been any reports of successful automated classification with the same level of accuracy. In this study, we attempted to construct a urinary sediment image classification model using deep learning, a well-known artificial intelligence method and neural network technique^11^. However, it is difficult to obtain a large number of human clinical specimens. To perform deep learning under such circumstances, we examined the type of image processing that would be desirable. Today, there are some diseases for which fewer than hundreds of cases have been collected worldwide^12,13^. In such diseases, diagnostic criteria have not been established, and diverse data cannot be collected.

Augmentation is the process of expanding image data by applying various transformations to images to create large amounts of new image data. Augmentation can increase the diversity of the data^14^. Thus, augmentation has been performed in studies using deep learning in various fields^15,16^, but it is not clear what kind of augmentation improves the performance of the model. In this study, we tried various augmentation methods to build a better deep learning model from a small dataset. By clarifying the optimal parameters for deep learning with a small number of images, it is expected that image classification using deep learning will be much easier to apply in clinical practice.

## Methods

### Collected image data

Images of urinary sediment crystals were collected from the Methods of Urinary Sediment Examination 2010 (Japan Society of Clinical Laboratory Technologists, 2010) and from past questions of the National Examination for Clinical Laboratory Technologists of Japan. Many of the collected urinary sediment crystal images had wide margins around crystals or multiple crystals in a single image. Therefore, from each urinary sediment crystal image, a single crystal was cropped such that it was located in the center of the image, and so that the margins were appropriate. Multiple crystal images cropped from the same image were stored as a "group"; if only one crystal appeared in an image, the one cropped crystal was considered a "group." Because crystals cut from the same single image have the same background, color, exposure, and other image conditions, the system may use factors such as background to classify the images. To avoid this, crystals cut from the same image were treated as a group. The types of urinary sediments collected were: magnesium ammonium phosphate crystals, bilirubin crystals, two calcium carbonate crystals, five calcium oxalate crystals, calcium phosphate crystals, cystine crystals, two uric acid crystals, and two ammonium urate crystals. Images with the same name but different shapes were divided into different categories, which were referred to as subcategories. The total number of images collected was 441 images, with 129 groups. A breakdown is presented in Table 1.

**Table 1:** Collected images.

Category	Images	Groups
Magnesium ammonium phosphate	29	12
Bilirubin	28	13
Calcium carbonate 1	27	6
Calcium carbonate 2	51	6
Calcium oxalate 1	58	15
Calcium oxalate 2	6	5
Calcium oxalate 3	20	3
Calcium oxalate 4	31	6
Calcium oxalate 5	3	3
Calcium phosphate	30	15
Cystine	43	18
Uric acid 1	22	7
Uric acid 2	11	3
Ammonium urate 1	28	1
Ammonium urate 2	54	16

These 129 groups were randomly then divided by crystal category in a 6:1:3 ratio for study, validation, and testing. Since ammonium urate 1 consisted of only one group, its 28 images were divided by this ratio. For categories with fewer than 10 groups, at least one group was reserved for validation, and the remaining groups were divided in a 2:1 ratio for study and testing. The process of dividing the 129 groups into training, validation, and testing was performed 10 times to create 10 test image sets.

### Image augmentation process

For augmentation, we used Python 3.8.5^17^ and the Augmentor 0.2.8 Python library^18^, which performs random image processing within a set range on a given image, and outputs a specified number of images. Image processing that Augmentor can perform includes: “random_distortion,” “random_contrast,” “random_color,” “random_brightness,” “rotate_random90,” “shear,” “flip_random,” “greyscale,” “zoom,” “skew,” and “randomcrop.” In this study, we performed augmentation of training images with various combinations of these image processes. The parameters of the image processing are specified in Table 2. When using Augmentor we specify the number of images we need and each function processes images with the probability specified for each. When one image is selected from source images, the probability that each feature is performed on the image is shown in Table 2. This Augmentor’s stochastic behavior makes output images reasonably distributed and scattered.

**Table 2:** Parameters of augmentor features.

Feature	Probability	Other parameters
Random_distortion	0.5	Grid_width = 2, grid_height = 2, magnitude = 5
Random_contrast	0.5	Min_factor = 0.5, max_factor = 3
Random_color	0.5	Min_factor = 0.5, max_factor = 3
Random_brightness	0.5	Min_factor = 0.5, max_factor = 0.5
Rotate_random90	0.5	
Shear	0.5	Max_shear_left = 10, max_shear_right = 10
Flip_random	0.5	
Greyscale	0.3	
Zoom	0.3	Percentage_area = 0.8, randomize_percentage_area = False
Skew	0.5	
Randomcrop	0.5	Percentage_area = 0.5, randomize_percentage_area = False

To observe the effect of augmentation, the same augmentation was applied to the images in the test image set for each of the 10 image sets; the augmented images were trained, validation was performed on the validation images in that image set, and the model was constructed. The model was then used to classify the test images in the image set and the accuracy and loss values were calculated. In this manner, we obtained 10 accuracy values and 10 loss values from 10 image sets.

### Deep learning and evaluation

Deep learning using the Keras 2.2.4 Python library^19^ which is an open-source neural network processing library, was used to evaluate the training and augmentation results. After augmentation, the training image was scaled down to 3600 pixels (60 × 60 pixels) and trained by Keras. The network used was VGG-16, developed by Simonyan et al.^20^ which was highly rated in the category classification section of the ImageNet Large Scale Visual Recognition Challenge (ILSVRC)^21^. Since the early days of deep learning, Rectified Linear Unit (ReLU) has been considered the best activation function^11^, so we used ReLU in this study. Finally, we used the softmax layer to adjust the output values such that the overall probability became 1.0. In addition, a dropout layer was constructed to mitigate overlearning and improve generalization performance. For the loss function, we chose categorical cross-entropy, which is suitable for multiclass classification, and is the standard for multiple data classification problems. For the optimization algorithm, we used Adam^22^, although several of these are available in Keras. The choice of these functions affects the prediction results, amount of computation, and computation speed^19^. The learning late was 0.01 and the batch size was 2048. The accuracy value is the percentage of test images correctly classified by the model. Because the test images were cut from different photos for both training and validation, the test images were unknown to the model in question, so the accuracy of the model’s predictions could be evaluated.

### Hierarchy of crystal categories

Thus far, as described in the section “Collected image data,” when the same crystal had different shapes, we treated them separately as subcategories and gave them different labels (“calcium_oxalate1,” “calcium_oxalate2,” “calcium_oxalate3,” etc.) when training them in Keras. However, differences in the shape of same substance crystals depend on the concentration and the concentration of various ions^10^, and are of little significance in the diagnosis of disease, so we then gave the same labels to the crystals of the same type, and trained and evaluated them in the same way. In other words, there were one to five subcategories of calcium oxalates, and all of them were given the label “calcium_oxalate” and input to Keras. The same was performed for the other crystal types. We then examined how the accuracy values would change if the subcategories were treated separately or if the subcategories were not distinguished. To evaluate the model accuracy, precision, recall, and F1-score were calculated, and the area under the curve (AUC) was calculated after the Receiver Operating Characteristic (ROC) curve was obtained. Since this is a multi-class classification, their macro-averages were used in the calculation^23^. We used scikit-learn 0.24.1^24^ for the calculation of metrics such as accuracy, precision, recall, and F1-score. We used seaborn 0.11.2^25^ and matplotlib 3.3.2^26^ for drawing ROC curves and calculating the AUC.

### Comparison with Xception model

To examine the correctness of the present method, we compared it to Xception, a deep neural network developed by Challot^27^. The learning late was 0.01 (same as VGG-16). When the batch size had been set to 2048 (same as VGG-16), a GPU memory error occurred, so it was set to 1536. We trained Xception on the same dataset and classified the test set images, comparing the results on precision, recall, and F1-score, in addition to accuracy. As in the previous section, the ROC curve was drawn, and the AUC was calculated.

### Correction using real-world appearance ratios

To correct the accuracy of the predictions to match the crystal occurrence ratios in the real world, we performed a literature search for studies addressing the eight classifications used in this study. We found that Jean et al. reported the ratio of the percentage of crystal appearance in 1603 urine samples from Ouagadougou, the capital of Burkina Faso^28^. Using this data, we corrected for the ratio of appearance in the real world and evaluated the corrected accuracy values.

## Results

### Consideration of augmentation process

**Figure 1:**
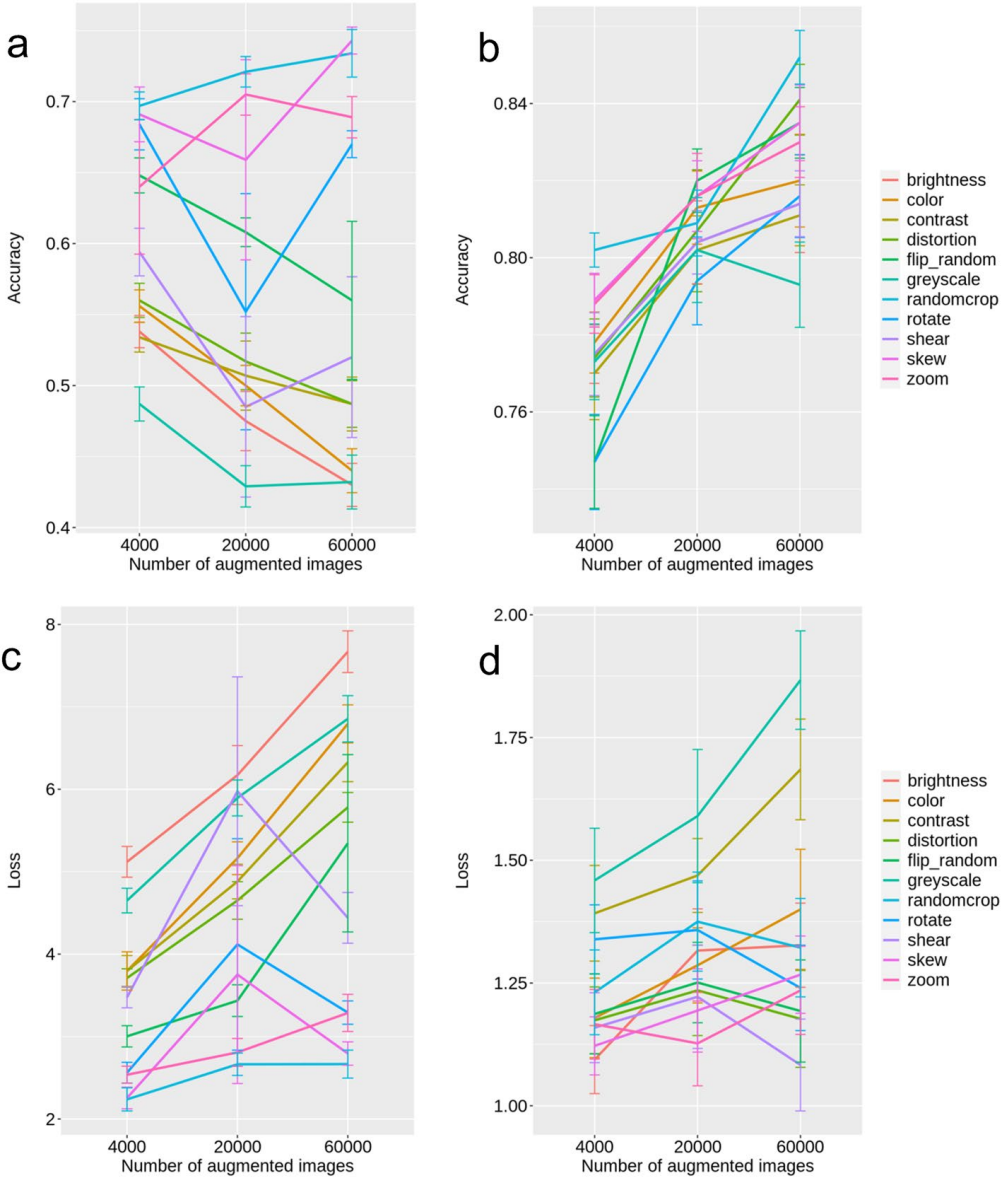
Accuracies and losses. (**a**) Accuracies by processing only one feature. (**b**) Accuracies by excluding one feature and processing the others. (**c**) Losses by processing only one feature. (**d**) Losses by excluding one feature and processing the others. Error bars indicate standard error of mean.

First, all 11 augmentation features were processed, and the average accuracy value was calculated; the accuracy value was 0.774 ± 0.010 (value ± standard error, same below) for 4000 augmented images, 0.811 ± 0.012 for 20,000 images, and 0.809 ± 0.020 for 60,000 images. This was not considered a sufficiently high classification accuracy. The evaluation of the 11 features was then performed individually, as shown in Fig. 1a,c. Comparing the results for the case of 4000 images and the case of 60,000 images, only three of them, “skew,” “randomcrop,” and “zoom,” showed a higher accuracy with 60,000 augmented images.

Therefore, we augmented the combination of these three parameters, “skew,” “randomcrop,” and “zoom,” and evaluated them in the same way; the accuracy value was 0.727 ± 0.008 for 4000 augmented images, 0.752 ± 0.015 for 20,000 images, and 0.757 ± 0.013 for 60,000 images. This was worse than that for learning using all the 11 features. This suggests that the accuracy tends to improve when more image features are used.

Next, we performed a learning pattern in which only one parameter was excluded and the others were used. The overall trend was that the accuracy tended to be higher for patterns that excluded one parameter than for patterns that only used one parameter in the augmentation process (Fig. 1a,b). In the pattern that excluded a single parameter, the higher the number of images obtained by augmentation, the higher the accuracy. The highest accuracy value was obtained in the pattern that excluded “randomcrop” and used the other features on 60,000 images, with an accuracy value of 0.852. The pattern that excluded “randomcrop” and used the other features obtained a higher accuracy value than that obtained for the other patterns when augmentation was performed on 4000 and 60,000 images, but was inferior to the patterns that excluded “zoom,” “skew,” or “flip_random” on 20,000 images. Interestingly, the pattern that excluded “randomcrop” and used the other features obtained a higher accuracy value than the pattern that used all 11 parameters when augmenting 60,000 images. Loss did not increase even when the number of images obtained by augmentation was increased, and we did not consider it to be a particular problem (Fig. 1c,d). The accuracy and loss values for these trials are available in Additional File 1.

Therefore, we decided that the best results would be obtained if the number of images generated by augmentation was set to 60,000 and the “randomcrop” pattern was excluded, which was used for the following analyses. The results of this pattern are presented in Table 3.

**Table 3:** Results for the pattern that excludes “randomcrop” and uses the other features.

	Validation accuracy	Validation loss	Accuracy	Loss
Average	0.826	1.851	0.852	1.322
Standard error	0.018	0.174	0.007	0.100
Max	0.914	2.681	0.877	1.765
Min	0.743	0.998	0.820	0.850

**Figure 2:**
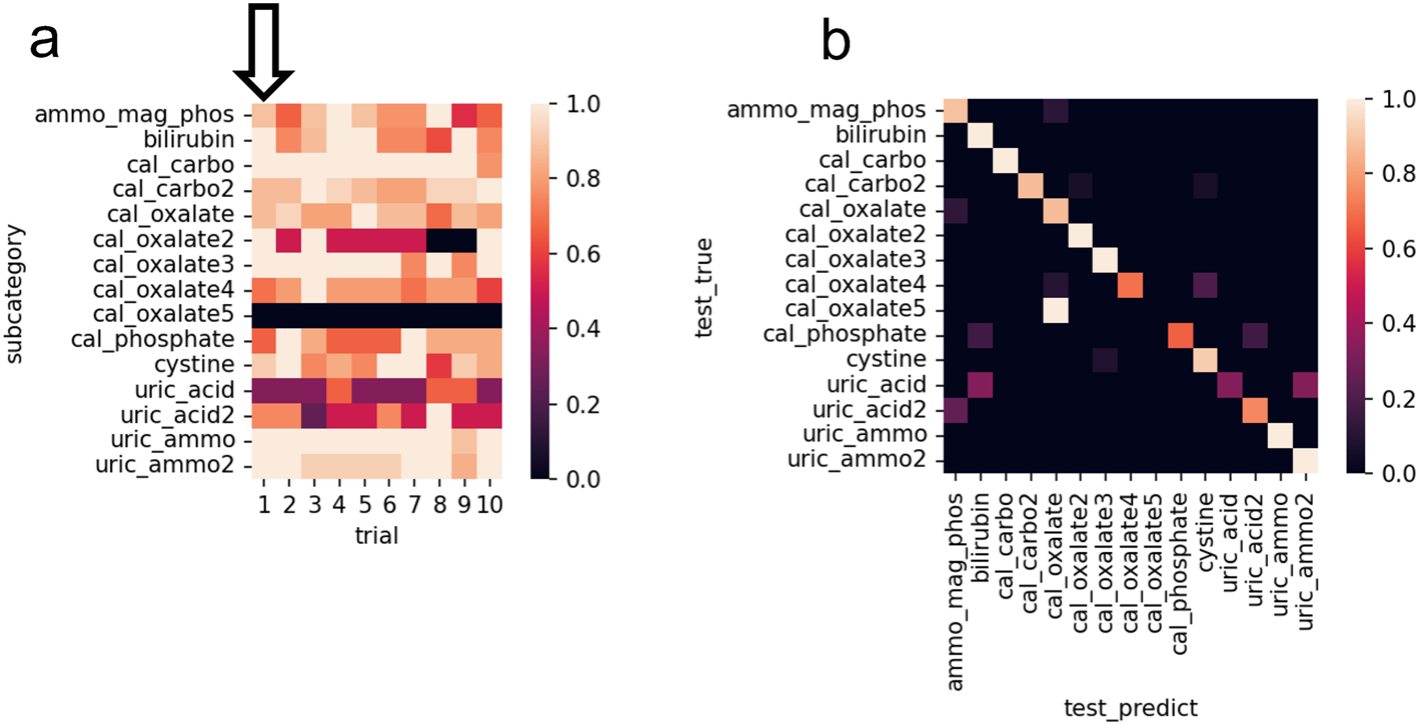
Result of 10 trials. (**a**) Correct answer rates. The arrow indicates the highest accuracy pattern (shown by **b**). Trials are listed from left to right in descending order of accuracy. (**b**) Relationships between the categories of correct answers and the categories predicted by the model for the highest accuracy pattern. Created with seaborn 0.11.2^25^. *ammo_mag_phos* magnesium ammonium phosphate, *cal_carbo* calcium carbonate, *cal_oxalate* calcium oxalate, *cal_phosphate* calcium phosphate, *uric_ammo* ammonium urate.

The percent correct values for each type of crystals in the 10 trials under the conditions described in the previous paragraph were summarized in a confusion matrix (Fig. 2a). For this evaluation, we used the set of test images that were pre-separated for each trial and not used for training. There were two trials that produced the highest accuracy value of 0.820. Among the two, for the trial with the smaller loss, a confusion matrix was created to visualize the relationship between the categories of correct answers and the categories predicted by the model (Fig. 2b). The prediction accuracy of calcium oxalate 5 was poor, which was the same for the other test sets. However, calcium carbonate and calcium oxalate 3 showed good accuracy. Those of other crystals, such as uric acid, were generally poor. We considered that a certain trend was observed for each category of crystal and that the differences due to repeated trials were lower than the differences due to category.

### Deep learning for hierarchical crystal categories

Subsequently, learning was conducted using upper-level categories without distinguishing between the crystal subcategories. The number of output images after augmentation processing for the training images was 60,000. The mean accuracy value when the subcategories were treated separately was 0.852 (Table 3), whereas the mean accuracy value in the case without distinguishing subcategories was 0.866 (Table 4).

**Table 4:** Results of not distinguishing subcategories.

	Validation accuracy	Validation loss	Accuracy	Loss
Average	0.869	1.277	0.866	1.132
Standard error	0.016	0.125	0.009	0.108
Max	0.923	1.858	0.918	1.705
Min	0.800	0.696	0.828	0.705

**Figure 3:**
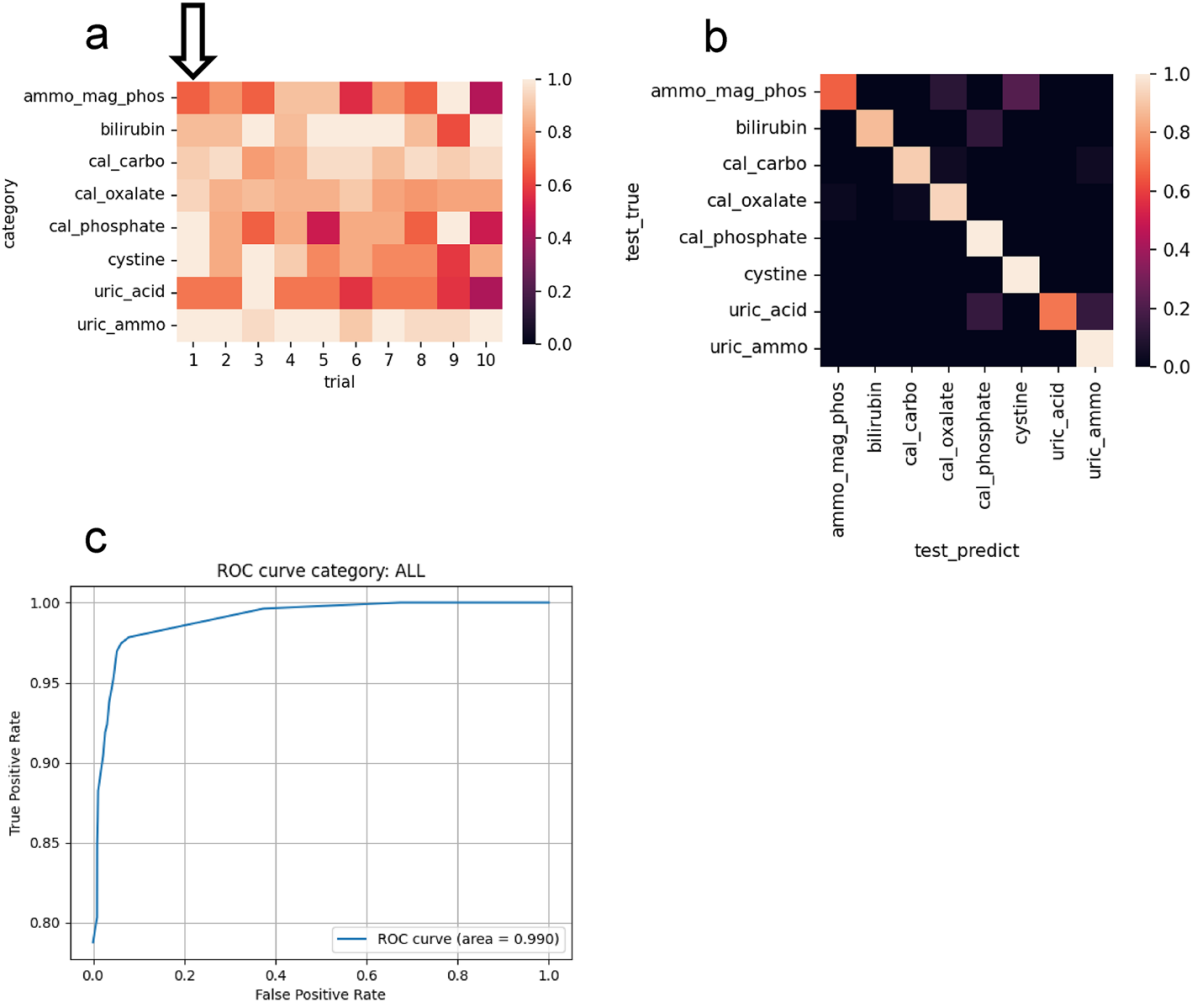
Result of 10 trials. (**a**) Correct answer rates. The arrow indicates the highest accuracy pattern [shown by (**b**) and (**c**)]. Trials are listed from left to right in descending order of accuracy. (**b**) Relationships between the categories of correct answers and the categories predicted by the model for the highest accuracy pattern. (**c**) ROC curve by the model for the highest accuracy pattern. Created with seaborn 0.11.2^25^ and matplotlib 3.3.2^26^. *ammo_mag_phos* magnesium ammonium phosphate, *cal_carbo* calcium carbonate, *cal_oxalate* calcium oxalate, *cal_phosphate* calcium phosphate, *uric_ammo* ammonium urate.

As well as distinguishing subcategories of crystals, the percent correct values for each type of crystals in the 10 trials under the conditions described in the previous paragraph were summarized in a confusion matrix (Fig. 3a). Similarly, for this evaluation, we used the set of test images that were pre-separated for each trial and not used for training. The trial with the highest accuracy value among the 10 test image sets was 0.918. A confusion matrix was created to visualize the relationship between the categories of correct answers and the categories predicted by the model (Fig. 3b), showing that within the 10 trials, there was a constant trend for each crystal type and that the difference between repeated trials was less than that between the different subcategories. The percentage of correct answers was high for calcium carbonate, bilirubin, and ammonium urate, but was low for magnesium ammonium phosphate and uric acid. The precision, recall, and F1-score were 0.910, 0.889, and 0.891, respectively. The ROC curves were drawn to evaluate the performance of the classifier (Fig. 3c), and the AUC was 0.990.

In summary, the best accuracy was obtained when all augmentation parameters except “randomcrop” were applied, the number of images to be generated was 60,000, and learning was performed without distinguishing subcategories. The trial with the highest accuracy value among the 10 test image sets was 0.918, exceeding the highest accuracy value of 0.877 when the subcategories were treated separately.

### Comparison with Xception model

**Figure 4:**
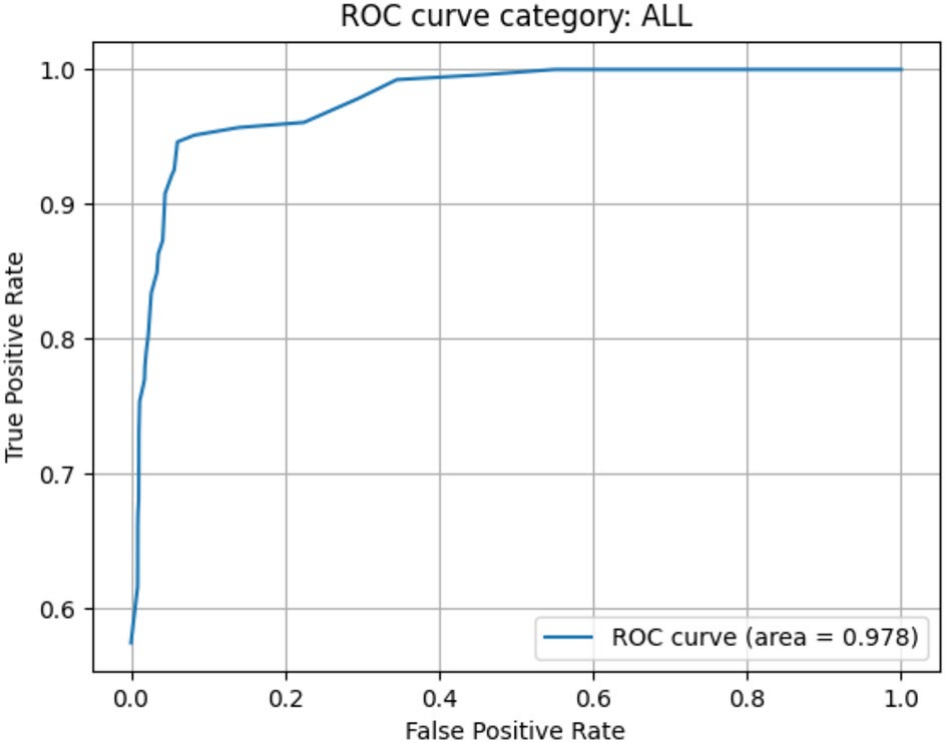
ROC curve by the Xception model. Created with matplotlib 3.3.2^26^.

We trained Xception model on the same dataset as the model that obtained the highest accuracy in the previous section and then classified the test set images. The accuracy, precision, recall, and F1-score were 0.844, 0.820, 0.813, and 0.808, respectively. The ROC curves were drawn (Fig. 4), and the AUC was 0.978.

### Correction using real-world appearance ratios

Using the ratio of the percentage of crystals appearing in 1653 urine samples from Ouagadougou, the capital of Burkina Faso, as reported by Jean et al.^28^ and weighting the accuracy percentage for each crystal in the pattern with the highest accuracy value (0.918), the following numerics are calculated in Table 5. The total weighted number of correct answers was 1462.258, and the ratio of the number of occurrences to the total number of samples was 0.885.

**Table 5:** Weighting the percentage of correct answers per crystal by occurrence ratio.

Category of crystal	Correct answer rate	Appearance ratio	Correct answer rate × appearance ratio
Magnesium ammonium phosphate	0.667	73	48.667
Bilirubin	0.875	11	9.625
Calcium carbonate	0.920	139	127.880
Calcium oxalate	0.939	932	875.515
Calcium phosphate	1.000	139	139
Cystine	1.000	6	6
Uric acid	0.714	341	1243.571
Ammonium urate	1.000	12	12
Overall	0.918	1653	1462.258

## Discussion

In this study, we attempted to build a model for classifying urinary sediment crystals by deep learning using collected images. All possible processes were performed in the augmentation of images, and better results were obtained when the number of images obtained by augmentation was large. However, better accuracy was obtained when random cropping was excluded from the augmentation processes. We speculate that this is because, unlike the other processes, random cropping could destroy the principle of one crystal per image. Regarding the number of images obtained by augmentation, within the scope of this study, the greater the number of images, the higher the accuracy value. However, because of graphics card memory shortage, it was difficult to further increase the number of images for training.

It was reported that the improvement in the accuracy with augmentation was 1.54%^15^. Although various conditions may be involved, in this study, increasing the number of images generated by augmentation sometimes reduced accuracy by a few percent to 10%. Therefore, it can be said that augmentation does not necessarily increase the percentage of correct answers; if one uses augmentation in research, it is necessary to consider the type of augmentation to be used.

Different subcategories of crystals have different apparent shapes, even if the crystals are composed of the same components. The same labels were given to crystals with different appearances, and yet the model could still identify the crystals without problems. Even if the images consisted of simple structures and looked different, the underlying features were extracted and learned. The model we obtained had a high F1 score and a high AUC, which was considered a better model.

Compared to later deep learning networks such as Xception, VGG-16 has a simple, classical architecture consisting only of basic elements such as convolutional layers, pooling layers, and dense layers. We considered that the results obtained by VGG-16 were superior to those of Xception, since detailed manipulations such as those in this study directry affect the teacher image.

Weighting based on the urinary sediment samples of Jean et al.^28^, the accuracy value was lower than that before weighting; this was probably due to the lower percentage of cystine, which had a high accuracy, and the higher percentage of ammonium urate, which had a low accuracy. Within the scope of our research, we were unable to find any other studies that reported the percentage of urinary sediment crystals in the eight categories discussed in this study. Therefore, it is debatable whether the population used here is appropriate, and in the future, it would be useful to examine crystals with high appearance ratios in the real world and try to improve the model for those crystals.

## Conclusions

In this study, a model was constructed by collecting images from textbooks and exam questions of national examinations in Japan. Although the number of images obtained was small in absolute terms for building a deep learning model, we obtained an accuracy of approximately 90% by testing various parameters, even with such a small number of images. In the augmentation of images, we obtained reliable results when all possible processes except “randomcrop” were performed and when the number of images obtained by augmentation was sufficiently large. The images used for training were images from textbooks which were used for initial training of beginner students, and the number of images used was sufficient to enable human learning. The fact that this level of accuracy was achieved even with such few images indicates the potential of artificial intelligence in the future. For future studies constructing deep learning models from real-world image data, in order to construct a better model, the parameters can be determined with reference to the parameter optimization performed in this study.

## Supplementary Information

Supplementary Information.

## Data Availability

All data generated or analyzed during this study are included in this published article and its supplementary information files.

## Abbreviations

ReLU: Rectified linear unit^11^
ROC: Receiver operating characteristic
AUC: Area under the curve
